# Radiofrequency catheter ablation combined with spironolactone in the treatment of atrial fibrillation: A single‐center randomized controlled study

**DOI:** 10.1002/clc.23659

**Published:** 2021-06-02

**Authors:** Weiwei Wang, Quanhe Chen, Feilong Zhang, Xuehai Chen, Zhe Xu, Xudong Sun, Jinguo Li, Lianglong Chen, Jianhua Chen

**Affiliations:** ^1^ Department of Cardiology Fujian Medical University Union Hospital & Fujian Provincial Institute of Coronary Disease Fuzhou China; ^2^ Union Clinic Medical College Fujian Medical University Fuzhou China

**Keywords:** aldosterone, atrial fibrillation, atrial remodeling, radiofrequency ablation, spironolactone

## Abstract

At present, the question of whether radiofrequency ablation (RFA) combined with spironolactone can reduce the levels of plasma angiotensin II (AngII) and aldosterone (ALD) in patients with atrial fibrillation (AF) and reduce the recurrence of AF has not been reported.

**Hypothesis:**

The present study evaluates the effect of spironolactone as an ALD antagonist on the short‐term and long‐term recurrence of AF after RFA. A total of 203 patients were enrolled in the present study, with 102 patients in the spironolactone therapy group (Group PVI/SP) and 101 patients in the control group (Group PVI alone). The AngII and ALD levels and the size of the left atrium in patients with AF were observed in order to evaluate the relationship between the combination therapy of spironolactone with RFA and the success rate in AF treatment. After therapy, the levels of AngII (52.8 vs. 64.3 pg/ml, p < .001), ALD (45.7 vs. 60.6 pg/ml, p = .016), and N‐terminal of B‐type natriuretic peptide (NT‐proBNP) (73.5 vs. 110 pg/ml, p = .016), along with the size of the left atrium (35.8 vs. 37.2 mm, p = .007), were all significantly lower in Group PVI/SP compared with Group PVI alone. The cumulative AF‐free survival rate was higher in Group PVI/SP than in Group PVI alone after treatment (85.3% vs.73.3%, p = .033). In RFA combined with spironolactone treatment, spironolactone can directly antagonize the effects of ALD and AngII and the recurrence of AF and improve left ventricular function.

## 1 INTRODUCTION

Atrial fibrillation (AF) is the most common sustained cardiac arrhythmia in adults, and AF catheter ablation is a well‐established treatment for the prevention of AF recurrences. The cornerstone of AF catheter ablation is the complete isolation of pulmonary veins by linear lesions around their antrum.^1^ Atrial electrical remodeling and structural remodeling are important mechanisms in the occurrence and persistence of AF.^2^ The activation of the renin–angiotensin–aldosterone system (RAAS) is involved in the structural remodeling of the atrium, primarily through the activation of angiotensin II (AngII) and aldosterone (ALD).^3^ A previous study has suggested that spironolactone, an ALD receptor antagonist, is able to reduce the level of ALD in patients with AF, as well as inhibiting atrial fibrosis, thus reducing the recurrence of AF.^4^ At present, the question of whether radiofrequency ablation (RFA) combined with spironolactone can reduce the AngII and ALD levels in patients with AF, and thus reduce the recurrence of AF, has not been reported. The present study is a single‐center prospective randomized study with the purposes of investigating the effect of spironolactone combined with RFA on the plasma AngII and ALD levels and the left atrium size in patients with AF and evaluating the relationship between this combination therapy and the success rate of AF treatment, so as to provide further clinical therapeutic investigation on increasing the success rate of AF treatment through the use of RFA procedures.

## 2 METHODS

### 2.1 Objects

The 379 patients with symptomatic AF treated by radiofrequency catheter ablation were enrolled. Among these patients, 215 met the inclusion criteria. The inclusion criteria were as follows: (1) patients with an age of ≥18 and ≤ 80 years old; (2) patients with paroxysmal or persistent AF (duration ≤12 months) who were to be treated by radiofrequency catheter ablation; (3) patients with grade I, II, or III New York Heart Association (NYHA) heart function before ablation; and (4) patients who had not been treated with spironolactone within 1 month prior to enrollment. The exclusion criteria were as follows: (1) patients with an age < 18 or > 80 years old; (2) patients with grade IV NYHA heart function before ablation; (3) patients with persistent AF lasting more than 12 months; (4) patients who did not achieve the intended surgical goals due to complications; (5) patients in whom AF continued after CA ablation and who were unable to convert to sinus rhythm after direct current synchronous cardioversion on the body surface, thus requiring additional ablation (such as linear ablation and fragment potential ablation) during CA ablation; (6) patients who could not follow up according to the specified timetable after the operation; (7) patients who were treated with spironolactone within 1 month prior to enrollment; and (8) patients who were treated with spironolactone within 1 month prior to enrollment.

The design of the present study was reviewed and approved by the Ethics Committee of the Affiliated Union Hospital of Fujian Medical University (approval No. 2011CXKT001). All patients signed an informed consent before RFA and agreed to receive follow‐up examinations in accordance with the study's design.

### 2.2 RFA methods

Local anesthesia was applied in all cases. The left and right femoral veins were punctured, and a 10‐electrode catheter was inserted through the left femoral vein to enter the coronary sinus. The atrial septum was punctured twice through the right femoral vein, and two 8F preface sheaths were sent into the left atrium. Through the preface sheaths, nonselective left and right pulmonary venography was performed to show the opening and line of each pulmonary vein. A 10‐pole annular catheter (Lasso, BioSense Webster) and 3.5 mm saline perfusion catheter (Thermo‐cool Navistar, BioSense Webster) were sent to the left atrium through the preface sheath. Under the guidance of the mapping system (CARTO 3, BioSense Webster), the Lasso catheter was used to construct a three‐dimensional structure of the left atrium. The Lasso catheter was sent into each pulmonary vein to measure the vein's potential. The position of each pulmonary vein opening and its potential characteristics were marked based on the three‐dimensional structure of the left atrium. The preset ablation power was within 25 ~ 35 W, and the temperature was set at 43°C. The perfusion volume of normal saline during ablation was 17 ml/min. Linear ablation was performed around the ipsilateral pulmonary vein, 5–10 mm away from the pulmonary vein opening. The electrical isolation of the pulmonary vein was the end point of ablation.

In patients with paroxysmal AF, the end point of the operation occurred either after the electrical isolation of the pulmonary vein with the intravenous injection of isoproterenol, which increased the patient's heart rate by 30% or more above the basal heart rate, or the confirmation that electrical conduction between the bilateral pulmonary vein and left atrium was not recovered, and 280–160 ms stimulation at the proximal end of the coronary sinus could not induce AF, atrial flutter (AFL), atrial tachycardia (AT), or other rapid atrial arrhythmias. In patients with persistent AF, if the AF was terminated and the sinus rhythm was restored after bilateral pulmonary vein isolation, the end point of the operation was the same as that in patients with paroxysmal AF. In patients with persistent AF, if the AF could not be terminated after bilateral pulmonary vein electrical isolation, 150 mg of amiodarone was injected intravenously, and the patient was then treated with direct current synchronous cardioversion with 150–200 J via the body surface. In patients who were able to maintain the sinus rhythm, the end point of the operation was the same as that in patients with paroxysmal AF. Linear ablation of the top of the left atrium, mitral annulus, and isthmus of the tricuspid annulus was necessary for patients who could not maintain sinus rhythm or whose AFL and AF could be induced by 280–160 ms stimulation of the proximal end of the coronary sinus; these patients were excluded.

### 2.3 Postoperative follow‐up

**FIGURE 1:**
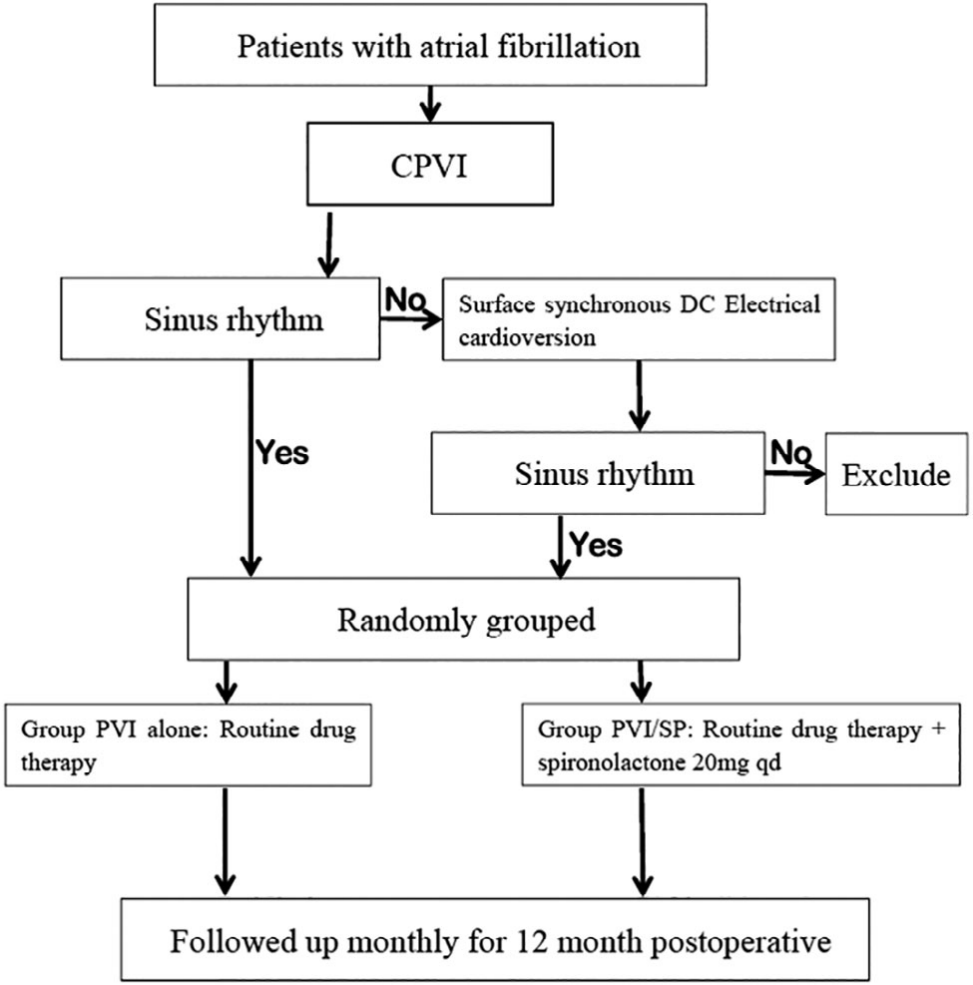
Flowchart of the patient's treatment follow‐up

All patients were followed up monthly for 12 months via outpatient visits or telephone calls. If the patient had any discomfort, he/she could also contact the doctor by telephone, information message, WeChat, etc. for additional follow‐up and to check the ECG and/or 24 h ambulatory electrocardiogram. A CA ablation was considered successful if the patient experienced no AF or tachycardia lasting more than 1 min during the follow‐up period. The first 3 months after CA ablation was designated as the blank period, during which a recurrence of AF, AFL, or AT was considered an early recurrence. A recurrence of AF, AFL or AT during the 3 to 12 months after ablation was considered a late recurrence. The levels of AngII, ALD, N‐terminal of B‐type natriuretic peptide (NT‐proBNP), and transthoracic echocardiography were monitored in all patients before and 3 months after CA ablation (Figure 1).

### 2.4 Drug therapy after CA ablation

Routine anticoagulant treatment was carried out for 3 months after CA ablation. After 3 months, the decision of whether to continue anticoagulant treatment was made jointly by the follow‐up physician and the patient based on the patient's CHA_2_DS_2_‐VASc score. The antiarrhythmic drugs propafenone or amiodarone were routinely used for 3 months after CA ablation. After 3 months, the antiarrhythmic drugs could either be stopped or continued depending on whether the patient had rapid atrial arrhythmia. If necessary, the follow‐up physician was free to treat the patient with the angiotensin‐converting enzyme inhibitor (ACE‐I) or an angiotensin receptor blocker (ARB).

### 2.5 Grouping of patients

The patients were randomly divided into two groups according to the order of treatment and the random number table method. The spironolactone treatment group (Group PVI/SP) was given 20 mg of spironolactone orally once a day for 3 months. The control group (Group PVI alone) was not given spironolactone.

### 2.6 Statistical analysis

All data analysis was based on the principle of intention analysis. The Kolmogorov–Smirnov test was used to verify whether the data followed the normal distribution. The normal distribution data were represented by $\bar{x}$± s, and a *t* test was used to compare the differences between the groups. The non‐normal distribution bias data were represented by the median (25%, 75%), and non‐parametric testing was used to compare the differences between the groups. The enumeration data were expressed in cases (%) and tested by χ^2^ or the Fisher exact probability method. The Kaplan–Meier survival analysis and the log‐rank test were used to evaluate the differences in postoperative AF‐free survival time. All analyses were processed by SPSS 22.0 statistical software, and p < .05 (bilateral) was considered statistically significant.

## 3 RESULTS

**FIGURE 2:**
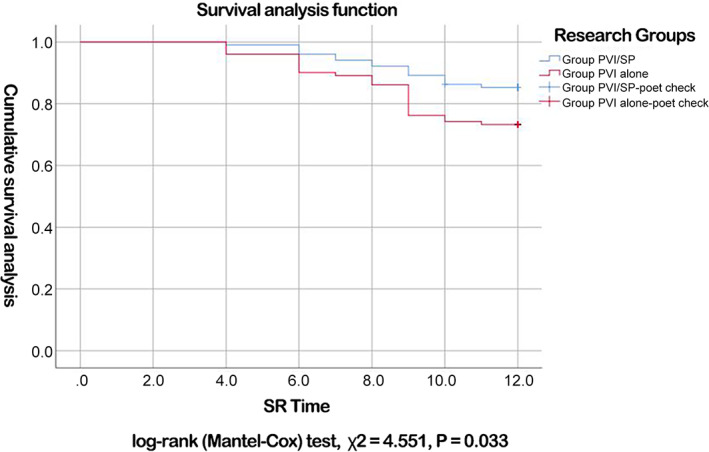
Comparison of cumulative atrial fibrillation‐free survival between two groups of patients

1. Baseline characteristics of the two groups: A total of 215 patients met the inclusion criteria, and all patients underwent catheter ablation. The 12 patients were excluded in accordance with exclusion criteria (5). During the twelve‐month follow‐up period, twelve patients were lost, and 203 patients completed the follow‐up. Group PVI/SP consisted of 102 patients, including 65 males (63.7%). In Group PVI/SP, 82 patients had paroxysmal AF (80.4%), and 20 had persistent AF. Thirty‐four cases (33.3%) were treated with ACE‐I or ARB. Group PVI alone consisted of 101 patients, including 65 males (64.4%). In Group PVI alone, 82 patients (81.3%) had paroxysmal AF, and 19 had persistent AF. Forty‐four (43.6%) cases were treated with ACE‐I or ARB. There was no significant difference in the baseline data between the two groups (Table 1).
2. Change in the AngII level in the two groups during follow‐up: There was no significant difference in the AngII level between Group PVI/SP and Group PVI alone (59.7 vs. 63.3 pg/ml, p = 0.182) before ablation. At 3 months after ablation, the AngII level had decreased significantly in Group PVI/SP compared with Group PVI alone (52.8 vs. 64.3 pg/ml, p < .001). The difference in the AngII level before and after CA ablation (ΔAngII) in Group PVI/SP was −6.2(−16.9–−0.5) pg/ml, and the difference was statistically significant (p < .001), while the ΔAngII in Group PVI alone was 1.0(−9.2–13.5) pg/ml (p = 0.279). The difference in ΔAngII between the two groups was statistically significant (p < .001) (Table 2).
3. Change in the ALD level in the two groups during follow‐up: There was no significant difference in the ALD level between Group PVI/SP and Group PVI alone before ablation (61.1 vs. 59.0 pg/ml, p = 0.829). At 3 months after ablation, the ALD level had decreased significantly in Group PVI/SP compared with Group PVI alone (45.7 vs. 60.6 pg/ml, p = .016). The difference in the ALD level before and after ablation (ΔALD) in Group PVI/SP was −11.4(−32.6, −1.1) pg/ml, p < .001, while the ΔALD in Group PVI alone was 1.5(−14.9, 12.4) pg/ml, p = 0.674. The difference in ΔALD between the two groups was statistically significant (p < .001) (Table 2).
4. Change in the NT‐proBNP level in the two groups during follow‐up: There was no significant difference in the NT‐proBNP level between Group PVI/SP and Group PVI alone before ablation (173.0 vs. 127.0 pg/ml, p = 0.194). At 3 months after ablation, the NT‐proBNP level had decreased significantly in Group PVI/SP compared with Group PVI alone (73.5 vs. 110.0 pg/ml, p = .016). The difference in the NT‐proBNP level before and after ablation (ΔNT‐proBNP) in Group PVI/SP was −87.0 pg/ml (p < .001), while the ΔNT‐proBNP in Group PVI alone was −21.00 pg/ml (p = .003). The difference in ΔNT‐proBNP between the two groups was statistically significant (p = .003) (Table 2).
5. Change in the size of the left atrium in the two groups during follow‐up: There was no significant difference in the size of the left atrium between Group PVI/SP and Group PVI alone before ablation (36.8 vs. 36.9 mm, p = 0.616). At 3 months after ablation, the size of the left atrium had decreased significantly in Group PVI/SP compared with Group PVI alone (35.8 vs. 37.2 mm, p = .007). The difference in the size of the left atrium size before and after CA ablation (ΔLA) in Group PVI/SP was −1.4(−2.5, −0.6) mm (*P* < .001), while the ΔLA in Group PVI alone was 0.5(−0.9, 1.4) mm (*P* = 0.665). The difference in the ΔLA between the two groups was statistically significant (p < .001) (Table 2).
6. Change in the LVEF in the two groups during follow‐up: There was no significant difference in the LVEF between Group PVI/SP and Group PVI alone before ablation (65.2% vs. 65.8%, p = 0.795). At 3 months after ablation, the LVEF had increased significantly in Group PVI/SP compared with Group PVI alone (67.5% vs. 64.7%, p *=* .009). The difference in the LVEF before and after CA ablation (ΔLVEF) in Group PVI/SP was 1.8(−0.3, 5.6)% (*P* < .001), while the ΔLVEF in Group PVI alone was 0.2(−3.4, 3.7)% (*P* = 0.566). The difference in the ΔLVEF between the two groups was statistically significant (p = .011) (Table 2).
7. Incidence of postoperative AF and analysis of AF‐free survival: During the first 3 months after CA ablation (the blank period), there were 19 (18.6%) and 31 (30.7%) patients with postoperative AF in Group PVI/SP and Group PVI alone, respectively. The difference between the two groups was statistically significant (p = .046). During the follow‐up period from 3–12 months after CA ablation, 15 (14.7%) and 27 (26.7%) patients in Group PVI/SP and Group PVI alone, respectively, had a recurrence of AF. The difference between the two groups was statistically significant (p = .039) (Table 3). The cumulative AF‐free survival rates in Group PVI/SP and Group PVI alone were 85.3% and 73.3%, respectively. In the log‐rank test, the χ^2^ was 4.551, with p = .033 (Figure 2). All patients with postoperative recurrence were treated with oral amiodarone. At the end of the 12‐month follow‐up, eight patients (53.3%) in group PVI/SP and 18 patients (66.7%) in group PVI alone were still receiving oral amiodarone, and there was no statistical significance between the two (p = .394).
8. Change in the mean pulmonary artery (PA) pressure in the two groups during follow‐up: There was no significant difference in the PA pressure between Group PVI/SP and Group PVI alone before ablation (28 vs. 28 mmHg, *P* = 0.752). At 3 months after ablation, the PA pressure had decreased significantly in Group PVI/SP compared with Group PVI alone (24 vs. 27 mmHg, p < .001). The difference in the PA pressure before and after surgery (ΔPA) in Group PVI/SP was −3.5(−7.0, 1.3) mmHg, p < .001 (−2.50–−0.60) mmHg, and this difference was statistically significant (p < .001), while the ΔPA in Group PVI alone was −1.0(−4.0, 2.1) mmHg (p = .067). The difference in the ΔPA between the two groups was statistically significant (p < .006) (Table 2).
9. Survival analysis by the Cox regression model revealed that spironolactone treatment (HR 0.337, 95% CI 0.157–0.726, p = .006), ALD level (HR 1.012, 95% CI 1.004–1.021, p = .006), gender (HR 2.482, 95% CI 1.053–5.853, p = .038), age (HR 1.068, 95% CI 1.015–1.123, p = .011), and ejection fraction (HR 0.933, 95% CI 0.873–0.997, p = .041) were correlated with the survival time with sinus rhythm after surgery in patients with AF. The type of AF, the left atrial diameter before ablation, and the level of plasma AngII were not correlated with survival time (Table 3).
10. Risk of recurrence of atrial fibrillation: ALD_D, ALD_D(1), ALD_(2), ALD_D(3) are grouped based on the ALD values in the 25%, 50%, 75%, and 100% percentile. Using group 0 as a criterion, which is the lowest ALD, group ALD_D(1), group ALD_D(2) and group ALD_D(3) had a 0, 0.112, and 0.275 increased risk of AF recurrence, respectively. This indicates a trend of increasing risk of AF recurrence with increasing ALD values. The trend test p trend = .031 suggests that this increasing trend is statistically significant.
11. During oral spironolactone treatment in Group PVI/SP, five male patients complained of mild breast pain. The patients were informed that the breast pain was a side effect of oral spironolactone and the symptoms would disappear after the medication was stopped. All five patients were treated with oral spironolactone for 3 months. The breast pain disappeared completely within 2 weeks of withdrawal. No side effects such as hyperkalemia and male mammary gland development were found in Group PVI/SP.

**TABLE 1:** Baseline characteristics of the two groups

	Group L(*n* = 102)	Grroup N(*n* = 101)	p value
Age (years‐old)	59.0(51.75,67.0)	60.0(52.0,66.0)	0.869
male (*n*,%)	67(65.7%)	65(64.4%)	0.843
Types of atrial fibrillation			
Paroxysmal atrial fibrillation (*n*,%)	82, 80.4	82, 81.3	0.886
persistent atrial fibrillation (*n*,%)	20, 19.6	19, 18.7	
BMI	24.3 ± 3.2	24.7 ± 3.3	0.320
Hypertension (*n*)	49	58	0.180
Coronary heart disease (*n*)	12	17	0.302
Diabetes mellitus (*n*)	16	18	0.684
NYHA			0.576
I (*n*)	70	62	
II (*n*)	30	36	
III (*n*)	2	3	
CrCl(ml/min)	74.8(63.5, 87.3)	75.0(62.0, 89.5)	0.956
CHA_2_DS_2_‐VASc score(*n*)			
0	16	11	
1	35	31	0.418
≥2	51	59	
HAS‐BLED score(*n*)			
0–2	88	80	0.183
≥3	14	21	
Smoking history (*n*)	34	32	0.802
TIA/ischemic stroke (*n*)	14	20	0.246
Use of ACE‐I or ARB (*n*)	34	44	0.960
Use of statins (*n*)	23	24	0.838
Use of β‐blockers (*n*)	13	16	0.528

**TABLE 2:** Change in AngII, ALD, NT‐proBNP level and in left atrium (LA) size in the two groups during follow‐up

		Group PVI/SP(*n* = 102)	Group PVI alone(*n* = 101)	p value
Ang II (pg/ml)	before ablation	59.7(51.3, 74.1)	63.3(51.3, 75.5)	0.182
	3 months after ablation	52.8(40.4, 63.1)	64.3(52.5, 80.7)	.000
	Δ AngII	−6.2(−16.9, −0.5)	1.0(−9.2, 13.5)	.000
ALD (pg/ml)	before ablation	61.1(37.1, 96.1)	59.0(36.5, 92.5)	0.829
	3 months after ablation	45.7(32.3, 73.3)	60.6(38.2, 85.2)	.016
	Δ ALD	−11.4(−32.6, ‐1.1)	1.5(−14.9, 12.4)	.000
LA (mm)	before ablation	36.8(33.5, 40.1)	36.9(32.7, 39.7)	0.616
	3 months after ablation	35.8(32.3, 38.3)	37.2(33.7, 39.4)	.007
	Δ LA	−1.4(−2.5, −0.6)	0.5(−0.9, 1.4)	.000
PA (mmHg)	before ablation	28(22, 33)	28(21, 33)	0.752
	3 months after ablation	24(20, 29)	27(21, 32)	.010
	Δ PA	−3.5(−7.0, 1.3)	−1.0(−4.0, 2.1)	.006
LVEF (%)	before ablation	65.2(62.3, 68.5)	65.8(62.0, 68.2)	0.795
	3 months after ablation	67.5(64.4, 70.2)	64.7(60.8, 69.2)	.009
	Δ LVEF	1.8(−0.3, 5.6)	0.2(−3.4, 3.7)	.011
NT‐ proBNP (pg/ml)	before ablation	173.0(70.3, 512.5)	127.0(58.5, 353.0)	0.194
	3 months after ablation	73.5(43.0, 142.5)	110.0(50.0, 233.0)	.016
	Δ NT‐proBNP	−87.0(−301.0, ‐9.8)	−21.0(−177.0, 19.5)	.003
Recurrence	The first 3 months after surgery	19(18.6)	31(30.7)	.046
	3–12 months after surgery	15(14.7)	27(26.7)	.039

**TABLE 3:** Parameter estimation for a fully variable model

	B	SE	Wald	Degree of freedom	Significance	Exp(B)	95.0% CI for Exp(B)	
							Lower	Higher
Grouping study	−1.087	0.391	7.707	1	0.006	0.337	0.157	0.726
Gender	0.909	0.438	4.318	1	0.038	2.482	1.053	5.853
Age	0.066	0.026	6.479	1	0.011	1.068	1.015	1.123
Types of atrial fibrillation	0. 592	0.509	1.353	1	0.245	1.808	0.666	4.908
ALD	0.012	0.004	7.578	1	0.006	1.012	1.004	1.021
ALD_D			8.886	3	0.031			
ALD_D (1)	−12.72	143.134	0.008	1	0.929	0.000	0.000	2.059E+116
ALD_D (2)	−2.191	0.841	6.785	1	0.009	0.112	0.022	0.581
ALD_D (3)	−1.289	0.546	5.575	1	0.018	0.275	0.094	0.803
Ang II	0.001	0.005	0.022	1	0.882	1.001	0.990	1.001
NT‐proBNP	−0.002	0.001	2.146	1	0.143	0.998	0.996	1.001
LA	0.048	0.044	1.202	1	0.273	1.049	0.963	1.144
LVEF	−0.069	0.034	4.181	1	0.041	0.933	0.873	0.997

*Note*: ALD_D, ALD_D(1), ALD_(2), ALD_D(3) are grouped based on ALD's Values in the 25%, 50%, 75%, and 100% percentile.

## 4 DISCUSSION

The results of this study showed that CPVI combined with spironolactone can reduce the levels of AngII and ALD, improve the maintenance rate of the sinus rhythm after catheter ablation, narrow the diameter of the left atrium, reduce the pulmonary artery pressure, and improve the function of the left ventricle.

AF catheter ablation is a well‐established treatment for the prevention of AF recurrences. When performed by appropriately trained operators, AF catheter ablation is a safe and superior alternative to AADs in maintaining sinus rhythm and improving symptoms. The cornerstone of AF catheter ablation is the complete isolation of pulmonary veins by linear lesions around their antrum, and more extensive ablation has been advocated for persistent AF. This may include: linear lesions in the atria; isolation of the LAA or the superior vena cava; ablation of complex fractionated electrograms, rotors, non‐pulmonary vein foci, or ganglionated plexi; fibrosis‐guided voltage; and/or MRI‐mapping, or ablation of high dominant frequency sites. However, any additional benefits of extensive ablation versus pulmonary vein isolation alone that justify its use during the first procedure, are yet to be confirmed.^1^

The transmural myocardial injury caused by catheter ablation must initiate the repair process of the myocardial injury. The scope of the injury repair increases with an increase in the size of the site of the ablation injury, and the process of damage repair is self‐limited. As long as the factors that cause the damage are removed, the repair will automatically terminate and will not continue to exist. If the ablation scope in the atrium is too great, the damage repair process will have an impact on atrial muscle fibrillation. All patients in this study underwent CPVI ablation without any additional ablation (e.g., linear ablation, matrix ablation, CFAES ablation or GP ablation). The degree of damage caused by atrial ablation and the degree of damage repair after ablation are comparable.

Renin‐angiotensin system (RAS) inhibitors significantly reduce the recurrence of atrial fibrillation in patients following catheter ablation (OR, 0.61; 95% CI, 0.45–0.82).^5^ The arrhythmogenic substrate of AF is driven by atrial fibrosis,^6^ and AF itself also promotes atrial fibrosis.^7^ ALD binding to the mineralocorticoid receptor and subsequent cardiac fibrosis formation is strongly associated with increased AF propensity.^8^ Neefs et al. demonstrated that the mineralocorticoid receptor antagonists (MRA), spironolactone, and eplerenone significantly reduce new‐onset AF and recurrent AF; MRA treatment can be considered an additive therapeutic strategy for AF.^9^ Alexandre et al. showed a significant overall reduction in AF occurrence in patients treated with MRA versus the control groups (OR 0.55; 95% CI, 0.44–0.70, p < .00001), with the greatest benefit shown with regard to recurrent AF episodes (OR 0.42; 95% CI, 0.31–0.59, p < .00001).^10^ The renin–angiotensin system (RAS) is involved in the genesis of arrhythmia by the following two mechanisms: the induction of atrial fibrosis and structural remodeling by mitogen‐activated protein kinase (MAPK) expression and a reduction of collagenase activity, and the induction of electrical remodeling by a shortening of the atrial effective refractory period (AERP) and the action potential duration.^11^

The structural remodeling of the atria manifested mainly as atrial fibrosis and atrial enlargement. The left‐atrial size is also an important predictor of the recurrence of atrial fibrillation after cardioversion treatment.^12^ The results of this study showed that in Group PVI alone, there was no difference in the AngII and ALD serum levels 3 months after CA ablation operation, and there was no difference in the size of the left atrium. This indicates that the repair process of the myocardial injury caused by catheter ablation had no effect on the process of atrial fibrosis, and the catheter ablation itself did not increase the atrial fibrosis.

One meta‐analysis by Zhao suggested that RAS inhibitors were of significant benefit in reducing the recurrence rate of AF after catheter ablation.^5^ Rienstra et al. showed that MRA therapy improves sinus rhythm maintenance in patients with persistent AF and mild‐to‐moderate heart failure.^13^ The AngII and ALD serum levels were significantly lower in Group PVI/SP 3 months after surgery than before surgery (both p < .001). Compared with Group PVI alone, the AngII and ALD serum levels were significantly lower in Group PVI/SP 3 months after surgery (all p < .05). Kimura et al. demonstrated that spironolactone improves atrial conduction and remodeling in patients with heart failure.^14^ Mayyas et al. posited that ALD antagonists may be more helpful in preventing the progression of AF than in suppressing the onset of AF; this suggests that ALD has a greater effect on AF substrate than triggers.^8^ Electrical isolation of the pulmonary veins (PVI) was found to prevent AF by effectively eliminating triggers, which remains the cornerstone for ablation treatment.^15^ Therefore, CPVI combined with spironolactone is expected to improve atrial fibrosis and reduce the recurrence rate of atrial fibrillation. The results of this study showed a significant decrease in the inner diameter of the left atrium in Group PVI/SP (p < .001), and this diameter was smaller than in Group PVI alone (p < .05). Compared with PVI u.s group, Group PVI/SP not only had patients with a lower early recurrence rate (18.6% vs. 30.7%, p = .046) but also patients with a lower late recurrence rate (14.7% vs. 26.7%, p = .039). This illustrates that CPVI with spironolactone therapy can decrease the AngII and ALD serum levels after radiofrequency ablation of atrial fibrillation patients, thus increasing the role of the inhibition of myocardial fibrosis and narrowing the diameter of the left atrium, which effectively reduces the early and late recurrence after radiofrequency ablation of atrial fibrillation.

Marrouche et al. showed that catheter ablation for atrial fibrillation in patients with heart failure (NYHA grade II, III, or IV, left ventricular ejection fraction of 35% or less) was associated with a significantly lower rate of a composite end point of death from any cause or hospitalization for worsening heart failure than with medical therapy (28.5% vs. 44.6%, HR, 0.62; 95% CI, 0.43 to 0.87; p = .007). Significantly fewer patients in the ablation group were hospitalized for worsening heart failure (20.7% vs. 35.9%; HR, 0.56; 95% CI, 0.37 to 0.83; p = .004). The median absolute increase in LVEF was 8.0% in the ablation group and 0.2% in the medical‐therapy group (p = .005).^16^ Li et al. demonstrate that the use of spironolactone improves the left ventricular diastolic function in patients with heart failure with preserved ejection fraction.^17^ The results of this study showed that in Group PVI alone, there was no difference in LVEF 3 months after CA compared with that before CA ablation (p = 0.692). Although the patients' LVEF could not be increased, the NT‐proBNP level decreased significantly (p = .002), which suggests that transcatheter radiofrequency ablation can improve the left ventricular function of patients with normal LVEF. However, in Group PVI/SP, the NT‐proBNP level was significantly reduced 3 months after radiofrequency ablation compared with that before surgery (p < .001), and the absolute decrease of NT‐proBNP was greater than that in Group PVI alone (p = .039). In Group PVI/SP, 3 months after radiofrequency ablation, the LVEF showed a marked increase, with a ΔLVEF of (1.8 [0.3, 5.6])%, p = .002). It is suggested that the absolute value of the increased LVEF and decreased NT‐proBNP in Group PVI/SP is greater on account of the improvement in the atrial remodeling after the treatment of spironolactone; this improvement in the atrial remodeling improves the left ventricular function of patients, which is shown as an increased LVEF and decreased NT‐proBNP serum level.

The limitations of the present study:

1. Although a prospective randomized study design was adopted for the present study, the sample size was small, and it involved data analysis from a single center, which could lead to a certain degree of bias.
2. In the present study, the therapeutic course of spironolactone was only 3 months in duration, and so it was impossible to evaluate the most appropriate therapeutic course for spironolactone after RFA.
3. The side effects of spironolactone, such as hyperkalemia and hyperplasia of the mammary gland, were not observed in the present study, which could not provide an analytical basis for the safety of spironolactone treatment.
4. The follow‐up method adopted in the present study involved regular follow‐ups plus telephone follow‐ups when necessary; thus, it is inevitable that a small number of asymptomatic AF events were missed. The most appropriate dosage and course of treatment and the safety of the combination of spironolactone and RFA for AF are questions that need to be addressed in future studies.

## 5 CONCLUSION

In the combination therapy of RFA and spironolactone, spironolactone can directly antagonize the effects of ALD and AngII, reverse the electrical and structural remodeling of the atrium caused by the above two factors, reduce the diameter of the left atrium and the occurrence of AF, and improve the left ventricular function in patients with AF.

## CONFLICT OF INTEREST

The authors declare that they have no competing interests.

## AUTHORS CONTRIBUTION

Weiwei Wang and Jianhua Chen conceived the idea and conceptualized the study. Feilong Zhang and Xuehai Chen collected the data. Xuehai Chen, Xudong Sun and Jinguo Li analyzed the data. Weiwei Wang and Jianhua Chen drafted the manuscript, then and Lianglong Chen reviewed the manuscript. All authors read and approved the final draft.

## Data Availability

The datasets generated during and/or analyzed during the current study are available from the corresponding author on reasonable request.

## References

[1] Hindricks G, Potpara T, Dagres N, et al. 2020 ESC guidelines for the diagnosis and management of atrial fibrillation developed in collaboration with the European Association of Cardio‐Thoracic Surgery (EACTS). Eur Heart J. 2021;42(5):373‐498. 10.1093/eurheartj/ehaa612. 32860505

[2] Mazzini MJ, Monahan KM. Pharmacotherapy for atrial arrhythmias: present and future. Heart Rhythm. 2008;5(6 Suppl):S26‐S31. 10.1016/j.hrthm.2008.01.023. 18456197

[3] Kirchhof P, Fabritz L. Of hammers and screws: rennin‐angiotensin‐aldosterone system inhibition to prevent atrial fibrillation in patients with hypertension. Eur Heart J. 2014;35(18):1169‐1171. 10.1093/eurheartj/ehu068. 24566798

[4] Zhang SH, Wang J, Jin TR, et al. The role of spironolactone in the metabolism of serum type I collagen in elderly patients with atrial fibrillation. Eur Rev Med Pharmacol Sci. 2014;18(19):2903‐2907. 25339485

[5] Zhao J, Chen M, Zhuo C, et al. The effect of renin‐angiotensin system inhibitors on the recurrence of atrial fibrillation after catheter ablation. Int Heart J. 2020;61(6):1174‐1182. 10.1536/ihj.20-346. 33191354

[6] Nattel S, Burstein B, Dobrev D. Atrial remodeling and atrial fibrillation: mechanisms and implications. Circ Arrhythm Electrophysiol. 2008;1(1):62‐73. 10.1161/CIRCEP.107.754564. 19808395

[7] Dzeshka MS, Lip GY, Snezhitskiy V, et al. Cardiac fibrosis in patients with atrial fibrillation: mechanisms and clinical implications. J Am Coll Cardiol. 2015;66(8):943‐959. 10.1016/j.jacc.2015.06.1313. 26293766

[8] Mayyas F, Alzoubi KH, Van Wagoner DR. Impact of aldosterone antagonists on the substrate for atrial fibrillation: aldosterone promotes oxidative stress and atrial structural/electrical remodeling. Int J Cardiol. 2013;168(6):5135‐5142. 10.1016/j.ijcard.2013.08.022. 23993726 PMC4062912

[9] Neefs J, Van den Berg NWE, Limpens J, et al. Aldosterone pathway blockade to prevent atrial fibrillation: a systematic review and meta‐analysis. Int J Cardiol. 2017;231:155‐161. 10.1016/j.ijcard.2016.12.029. 28062142

[10] Alexandre J, Dolladille C, Douesnel L, et al. Effects of mineralocorticoid receptor antagonists on atrial fibrillation occurrence: a systematic review, meta‐analysis, and meta‐regression to identify modifying factors. J Am Heart Assoc. 2019;8(22):e013267. 10.1161/JAHA.119.013267. 31711383 PMC6915291

[11] Novo G, Guttilla D, Fazio G, et al. The role of the renin‐angiotensin system in atrial fibrillation and the therapeutic effects of ACE‐is and ARBS. Br J Clin Pharmacol. 2008;66(3):345‐351. 10.1111/j.1365-2125.2008.03234.x. 18782141 PMC2526238

[12] Mujović N, Marinković M, Lenarczyk R, et al. Catheter ablation of atrial fibrillation: an overview for clinicians. Adv Ther. 2017;34(8):1897‐1917. 10.1007/s12325-017-0590-z. 28733782 PMC5565661

[13] Rienstra M, Hobbelt AH, Alings M, et al. Targeted therapy of underlying conditions improves sinus rhythm maintenance in patients with persistent atrial fibrillation: results of the RACE 3 trial. Eur Heart J. 2018;39(32):2987‐2996. 10.1093/eurheartj/ehx739. 29401239

[14] Kimura M, Ogawa H, Wakeyama T, et al. Effects of mineralocorticoid receptor antagonist spironolactone on atrial conduction and remodeling in patients with heart failure. J Cardiol. 2011;57(2):208‐214. 10.1016/j.jjcc.2010.11.006. 21185153

[15] Boersma L, Rienstra M, Groot JR. Therapeutic options for patients with advanced atrial fibrillation: from lifestyle and medication to catheter and surgical ablation. Neth Heart J. 2020;28(Suppl 1):13‐18. 10.1007/s12471-020-01447-5. 32780326 PMC7419415

[16] Marrouche NF, Brachmann J, Andresen D, et al. Catheter ablation for atrial fibrillation with heart failure. N Engl J Med. 2018;378(5):417‐427. 10.1056/NEJMoa1707855. 29385358

[17] Li S, Zhang X, Dong M, et al. Effects of spironolactone in heart failure with preserved ejection fraction: a meta‐analysis of randomized controlled trials. Medicine (Baltimore). 2018;97(35):e11942. 10.1097/MD.0000000000011942. 30170387 PMC6392615

